# Audibility threshold for high frequencies inchildren with medical history of multiples episodes of bilateral secretory otitis media

**DOI:** 10.1016/S1808-8694(15)31071-5

**Published:** 2015-10-22

**Authors:** Mônica de Sá Ferreira, Katia de Almeida, Ciríaco Cristóvão Tavares Atherino

**Affiliations:** 1 Master's degree on speech therapy. Veiga de Almeida University. Speech therapist. Teacher of the graduation course on audiology at the Estacio de Sa University.; 2 Doctoral studies on human communication disorders - speech therapy. Sao Paulo Federal University. Adjunct professor in the speech therapy course at the Santa Casa de Sao Paulo Medical Science School and the professional master's degree course on speech therapy at the Veiga de Almeida University.; 3 Doctoral studies on otorhinolaryngology. Sao Paulo University. Doctor and adjunct professor of the otorhinolaryngology discipline at the Rio de Janeiro State University and the professional master's degree course on speech therapy at the Veiga de Almeida University. Head of the Otorhinolaryngology unit of the Ipanema General Hospital, Ministry of Health.; 4 Veiga de Almeida University.

**Keywords:** audiometry, children, secretory otitis media

## Summary

Relatively poorer audibility threshold for high frequency was found in children with medical history of multiples episodes of secretory otitis media. **Aim:** to characterize the audibility threshold for high frequencies in normal-hearing children with medical history of multiples episodes of bilateral secretory otitis media. **Materials and methods:** a sample of 31 children, from both genders, was divided in two groups: 14 subjects who had not more than 3 episodes of bilateral secretory otitis media (Group 1) and 17 subjects that experienced at least 4 episodes of this condition (Group 2). Pure-tone air conduction audiometry was tested at frequencies 9,000 to 18,000 Hz. **Study design:** transversal prospective. **Results:** there was no difference between audibility thresholds comparing right and left ears of subjects of both Group 1 and Group 2 in all tested frequencies. However, there was difference between audibility thresholds between subjects of Group 2 compared to Group 1 in all tested frequencies. **Conclusion:** 1- There was an increase in audibility thresholds with the increase in frequency. 2- High frequency audiometry separates subjects with history of at least four episodes of secretory otitis media, suggesting that these episodes are sufficient to promote statistically significant difference in high frequency thresholds.

## INTRODUCTION

Hearing loss, characterized by total or partial loss of hearing, may be classified in different severity levels. Similar to visual impairment, hearing loss may affect the full development of a person, and may interfere on learning.

A child with hearing loss is recognized when he or she does not respond to calls at a distance, when that child constantly asks for speech to be repeated, or constantly increases the volume of radio or television. Such a child is inattentive, changes letters in speech or in writing, and has learning difficulties at school.^1^

Otitis media is one of the most frequent health problems in childhood. Shorter duration of breastfeeding, early entrance to nurseries, and remaining close to a large number of children in the same nursery/school are some of the factors that have increased the incidence of this disease.^2^,^3^ Other causes, according to Hirata,4 are houses with fewer rooms and many people sleeping in the same place, the habit of pacifier or finger sucking, and passive smoking.

Pure tone audiometry is the traditional method for assessing auditory sensitivity between 250 and 8000Hz. There are cases, however, in which conventional frequencies are not sensitive to middle ear alterations, such as those that take place in chronic otitis media; this is an obstacle against a precise diagnosis. In certain conditions an audiogram with a sloping configuration is found, suggesting loss at higher frequencies.

Although some trials^35^,^36^ have reported loss of conductive hearing, others have shown that the basal turn of the cochlea is affected by chronic otitis media, which characterizes sensorineural hearing loss. High frequency audiometry was the test tool in many of these trials, which was very useful for the diagnosis, as this test provides an early assessment of the state of the cochlea at 9000 to 20000Hz (the upper limit of human perception).

Studies on the application and use of high frequency audiometry began around 1960, at a time when assessing frequencies between 300 and 8000Hz was considered sufficient to predict hearing at higher frequencies. Since then many authors have demonstrated the importance of high frequency audiometry to monitor ototoxicity and hearing loss induced by high sound pressures. A number of papers have also been published showing increased auditory thresholds at higher frequencies in cases of multiple episodes of otitis media with effusion (Dieroff & Schulmann,5 Mair, Fjermedal & Laukli,6 Sorri & Rantakallio,7 Rahko et al.,8 Mutlu et al.,^9^ and Margolis, Saly & Hunter^10^).

The aim of this trial was to compare high frequency auditory thresholds in children and the number of events of bilateral otitis media with effusion.

## REVIEW OF THE LITERATURE

Based on histopathology, Huang, Dulon & Schacht11 and Van Cauwenberge, Watelet & Dhooge^12^ showed that bacterial toxins and cytotoxins diffuse from the middle ear to the cochlea through the membrane of the round window in cases of otitis media. According to Winter et al.^13^ and Tuomanen,14 this would result in ultrastructural injury to the inner ear and rupture of cochlear membranes.

High frequency sensorineural hearing loss frequently coexists with otitis media with effusion or chronic otitis media.15,9 To analyze this phenomenon, Huang, Dulon & Schacht11 created experimental models of middle ear infection for electrophysiological and pathological studies of the cochlea. These authors stated that sensorineural hearing loss, whether permanent or not, could result from inflammation of the middle ear. They found that outer hair cells in the base of the cochlea were injured in cases of otitis media, and that there was evidence of increased round window membrane permeability for toxic substances originating in the middle ear.

Published papers in the literature have shown a correlation between middle ear diseases and language delay and disorders, and a loss of correlated abilities.

Petinou et al.^16^ demonstrated that children aged between 1 and 3 years, diagnosed with hearing loss, show increased language acquisition difficulties, decreased perception of speech sounds containing voiceless or fricative consonants such /s/ and /z/, and frequent phonetic errors when pronouncing /l/ and /r/.17 According to the authors the most common cause is mild conductive hearing loss - which may be unilateral - resulting from otitis media. Paradise18 noted that during these infections sound stimuli are distorted, which could explain the phonetic errors.

In a review Balbani & Montovani^19^ concluded that the main consequences of otitis media and hearing loss over language in these children are phonetic errors, speech articulation disorders, and reading comprehension difficulties.

Some authors have reported that language delays and disorders - and disorders of related abilities - are indicative of subsequent difficulties with reading and written language, which is based on oral language.^20^

To this date studies on audiologically normal individuals have shown that children have excellent auditory sensitivity to high frequency sounds.^21^, ^22^, ^23^

Pedalini & Sanchez^23^ investigated high frequency auditory thresholds in 158 subjects aged between 4 and 60 years (87 women and 71 men) to establish a mean threshold value in otologically normal individuals. The study included subjects with normal otoscopy and normal thresholds on conventional audiometry up to 25dB NA in both ears. The sample was divided into 6 groups according to age: 1st - 4 to 10 years; 2nd - 11 to 20 years; 3rd - 21 to 30 years; 4th - 31 to 40 years; 5th - 41 to 50 years; and 6th - 51 to 60 years. A Madsen model ORBITER OB922 audiometer with Sennheiser HDA 200 earphones was used to measure thresholds at 10000, 12500, 14000, and 16000Hz; results were given in dB NA. Results showed that thresholds were maintained up to 25dB in the first three groups (ages 4 to 30 years). In the fourth decade the threshold fell only at 16000Hz. In the fifth decade, thresholds fell at 12500, 14000, and 16000Hz, in that order. Finally, in the sixth decade, thresholds fell at all frequencies tested. The authors concluded that normal thresholds adopted for pure tone audiometry cannot be the same as those used for high frequencies.

Difficulties found when carrying out high frequency audiometry include earphone placement, which is significant due to resonance and stationary waves at frequencies over 15000Hz. This may result in significant individual variability, as half the wavelength is enough to produce resonance transversally to the external auditory canal. It is not uncommon to find threshold variations of 15 to 20dB in these cases.^24^, ^25^, ^26^

Various studies^27^, ^28^, ^29^, ^30^, ^31^, ^32^,^23^,^33^,^34^ have attempted to standardize auditory thresholds at frequencies over 8000Hz, correlating findings with gender and age group to establish normal values. Most papers agree that factors such as age and frequency at which auditory acuity is determined interfere on results. A further difficulty was that the size and configuration of the external acoustic canal varies significantly from person to person, which complicates the calibration of earphones used for high frequency audiometry. Improvements in earphone design and detailed knowledge of mechanical and acoustic concepts currently allow this test to be used routinely in audiological clinical practice.

Hunter et al.^37^ associated high frequency hearing loss with multiple episodes of otitis media with effusion after treatment. They concluded that two or more documented episodes were enough to raise high frequency auditory thresholds compared to a control group.

A similar study by Laitila et al.^38^ also found threshold differences in children with a history of otitis media; this difference was higher at high frequencies in children with 8 or more documented episodes.

Although there are doubts concerning the irreversibility of cochlear injury in otitis media, Mutlu et al.^9^ noted that some children with sensorineural high frequency hearing loss resulting from otitis media with effusion showed reversion of hearing loss following medical or surgical treatment.

## MATERIAL AND METHOD

This protocol, numbered 13/04, was submitted and approved by the Research Ethics Committee of the Veiga de Almeida University. Parents or caretakers were asked to allow participation of their children in this trial. Those that agreed signed a free informed consent form.

Our sample included 31 children of both genders aged between 7 and 12 years that were seen at a private clinic in the city of Petropolis, Rio de Janeiro state, between March 2004 and February 2005 and referred by otorhinolaryngologists. Fourteen children (6 male and 8 female) presented up to three episodes of bilateral otitis media with effusion (Group 1), and 17 children (9 male and 8 female) presented four or more episodes of bilateral otitis media with effusion (Group 2).

Exclusion criteria were children with thresholds over 15dB NA40 on conventional audiometry, a family history of congenital hearing loss, a history of systemic diseases such as diabetes, high blood pressure, cardiac disease, multiple sclerosis, or kidney failure, uninterrupted use of ototoxic drugs for over ten days, and prolonged exposure to high levels of sound pressure or acoustic trauma.

Children that were included had type A41 tympanometry in both ears.

A Grason Stadler GSI 61 audiometer calibrated according to international standards was used to carry out audiograms at 250 to 20000Hz. Telephonics TDH 50P earphones were used for conventional audiometry (250 to 8000Hz), and Sennheiser HDA-200 earphones were used for high frequencies (9000 to 18000Hz). Both tests were done in an acoustically treated booth according to ISO 8253-1.50

The test battery was done in 2 days to avoid fatigue and the interference of learning on results. A clinical history was taken on the first day, followed by meatoscopy, pure tone air conduction thresholds at 250 to 8000Hz, measurements of acoustic immitance, and training for high frequency threshold measurements (Sahyeb, Costa Filho & Alvarenga^34^). On the second day high frequency thresholds were investigated. Audiometric earphones were placed and pure tone thresholds were confirmed to minimize variability that might interfere with high frequency audiometry results.

Statistical analysis was done using the bicaudal paired Student's t-test to check the difference between means of auditory thresholds (dB NA) for ears in the same group, following the sample normality test (Kolmogorov-Smirnov). Comparison between auditory thresholds (dB NA) in the same ear for both groups was done using the bicaudal Student's t-test for samples with different variances.

The trend for auditory thresholds at all investigated frequencies was calculated by linear regression (line adjustment) to quantify the audiometric profile.

All of the statistical tests were calculated using the Statistical Package for the Social Sciences (SPSS) software and Microsoft® Office Excel 2003. The significance value (p) was 0.05.

## RESULTS AND DISCUSSION

Auditory thresholds in children included in Group 1 were never over 25dB NA at any frequency between 9000Hz and 18000Hz (Tables 1 and 2).

**Table 1:** Right and left ear auditory thresholds (dB NA) at 9000 to 18000Hz in males aged between 7 to 12 years in Group 1.

Grorp 1.														
Age	9.000		10.000		11.200		12.500		14.000		16.000		18.000	
	RE	LE	RE	LE	RE	LE	RE	LE	RE	LE	RE	LE	RE	LE
7	10	5	5	5	10	10	10	10	15	15	10	15	15	15
8	5	5	5	5	10	10	10	10	10	15	10	10	10	10
9	10	10	10	10	10	10	15	15	15	15	15	15	15	15
10	10	10	10	10	10	10	5	5	5	5	5	5	10	10
11	10	10	5	5	5	5	5	5	10	10	15	10	10	15
12	5	5	0	0	0	5	5	5	5	10	10	10	10	10
Mean	8,3	7,5	5,8	5,8	7,5	8,3	8,3	8,3	10	11,7	10,8	10,8	11,7	12,5

**Table 2:** Right and left ear auditory thresholds (dB NA) at 9000 to 18000Hz in females aged between 7 to 12 years in Group 1.

Group 1.														
Age	9.000		10.000		11.200		12.500		14.000		16.000		18.000	
	RE	LE	RE	LE	RE	LE	RE	LE	RE	LE	RE	LE	RE	LE
7	15	15	10	10	10	15	15	15	15	15	10	15	15	15
8	10	10	10	10	10	10	15	15	10	15	15	10	10	10
8	5	10	10	5	5	15	10	10	10	10	15	10	10	10
9	10	10	10	10	15	10	5	10	10	10	15	5	10	10
10	10	10	5	0	10	5	5	5	5	5	10	10	10	15
11	10	10	10	10	10	10	10	10	15	15	15	15	15	15
12	10	5	5	5	5	5	10	10	10	10	10	10	10	20
12	10	10	10	5	5	5	5	10	10	10	15	10	15	15
Mean	10	10	8,8	6,9	8,8	9,4	9,4	10,6	10,6	11,3	13,1	10,6	11,9	13,8

These findings are similar to those found in the literature, as demonstrated by Northern et al.,^27^ Mutlu et al.,^9^ Azevedo & Iorio,^32^ Margolis, Saly & Hunter,^10^ and Pedalini & Sanchez.^23^

In a sample containing 158 subjects aged between 4 and 60 years, Pedalini & Sanchez^23^ found that auditory thresholds were maintained up to 25dBNA in younger subjects aged between 4 and 30 years.

There were no statistically significant differences between right and left ear auditory thresholds in Group 1 at all of the frequencies we tested (Table 3).

**Table 3:** Auditory thresholds (dB NA) at 9000 to 18000Hz for right and left ears in Group 1 (values shown as the mean □ standard deviation [median]).

Frequency	Right ear	Left ear
9.000	9,3 ± 2,7 [10]	8,9 ± 2,9 [10]
10.000	7,5 ± 3,3 [10]	6,4 ± 3,6 [5]
11.200	8,2 ± 3,7 [10]	8,9 ± 3,5 [10]
12.500	8,9 ± 4,0 [10]	9,6 ± 3,7 [10]
14.000	10,4 ± 3,7 [10]	11,4 ± 3,6 [10]
16.000	12,1 ± 3,2 [12,5]	10,7 ± 3,3 [10]
18.000	11,8 ± 2,5 [10]	13,2 ± 3,2 [15]

In Group 2 (Tables 4 and 5) we found that auditory thresholds at 9000Hz to 18000Hz reached over 25dBNA at frequencies over 14000Hz. These thresholds were higher compared to those found in Group 1. Similar findings are seen in papers published by Lopponen et al.,^36^ Sorri, Maki-Torkko & Alho,^39^ and Laitila et al.^38^

**Table 4:** Auditory thresholds (dB NA) for right and left ears at 9000 to 18000Hz in males aged between 7 to 12 years in Group 2.

Group 2.														
Age	9.000		10.000		11.200		12.500		14.000		16.000		18.000	
	RE	LE	RE	LE	RE	LE	RE	LE	RE	LE	RE	LE	RE	LE
7	15	15	15	15	20	20	15	20	20	15	15	20	20	20
7	20	20	15	15	15	15	15	20	20	20	20	25	25	25
8	15	10	10	15	20	20	20	15	15	20	20	15	20	20
9	10	10	10	10	15	15	15	20	20	25	25	25	25	25
10	5	10	5	15	15	20	15	15	20	20	25	35	20	35
10	5	5	10	10	10	10	10	10	10	10	10	10	10	10
11	15	15	15	20	15	15	20	20	25	30	20	30	20	35
12	10	10	10	15	10	10	10	15	15	20	25	25	25	30
12	20	20	20	20	25	25	25	20	20	20	25	30	30	30
Mean	12,8	12,8	12,2	15	16,1	16,7	16,1	16,1	18,3	20	20,6	23,9	21,7	25,6

**Table 5:** Auditory thresholds (dB NA) for right and left ears at 9000 to 18000Hz in females aged between 7 to 12 years in Group 2.

Group 2.														
Age	9.000		10.000		11.200		12.500		14.000		16.000		18.000	
	RE	LE	RE	LE	RE	LE	RE	LE	RE	LE	RE	LE	RE	LE
7	15	15	15	15	20	20	25	25	30	25	35	25	25	30
8	10	10	15	20	15	15	20	20	20	25	30	30	30	25
8	10	15	15	15	15	15	25	25	25	25	35	30	25	30
9	10	10	10	10	15	15	15	15	15	15	20	20	25	25
9	15	15	15	15	15	15	20	20	20	20	20	20	20	25
10	15	5	15	15	15	20	20	20	20	15	25	25	25	25
11	20	20	25	20	25	20	25	25	30	30	40	40	35	35
12	15	15	15	15	15	15	20	20	20	15	20	20	20	20
Mean	13,8	13,1	15,6	15,6	16,9	16,9	21,3	21,3	22,5	21,2	28,1	26,3	25,6	26,9

There were no statistically significant differences between right and left ear auditory thresholds in Group 2 (Table 6). An explanation might be the history of bilateral otitis media with effusion, showing that previous otological disease results in higher thresholds and no difference between ears.^6^

**Table 6:** Auditory thresholds (dB NA) at 9000 to 18000Hz for right and left ears in Group 2 (values shown as the mean □ standard deviation [median]).

Frequency(Hz)	Right ear	Left ear
9.000	13,2 ± 4,7 [15]	12,9 ± 4,7 [15]
10.000	13,8 ± 4,5 [15]	15,3 ± 3,3 [15]
11.200	16,5 ± 4,2 [15]	16,8 ± 3,9 [15]
12.500	18,5 ± 4,9 [20]	19,1 ± 4,0 [20]
14.000	20,3 ± 5,1 [20]	20,6 ± 5,6 [20]
16.000	24,1 ± 7,5 [25]	25,0 ± 7,3 [25]
18.000	23,5 ± 5,5 [25]	26,2 ± 6,5 [25]

The absence of statistically significant differences between ears has also been reported in other papers, such as those by Frank & Dreisbach^45^, Hallmo, Borchgrevink & Mair,^43^ and Mattews et al.^44^

This result is not common to all papers. Although Sayeb et al.^34^ found increased auditory sensitivity to the left at all the frequencies they studies, the difference was not statistically significant on the chosen method (ANOVA). On the other hand Lopponen et al.,^36^ Hunter et al.,^37^ and Margolis, Saly & Hunter^10^ found statistically significant differences. Zeigelboim, Martinho & Marques^46^ found statistically significant differences only at 11200Hz in females.

The methodology used for audiometric assessment justifies high standard deviation values. 5dB steps of intensity do not allow a more precise evaluation of auditory thresholds. Vanden^47^ suggests that 1dB steps increase sensitivity. We suggest that trials be done using smaller intensity steps (1dB) to standardize high frequency auditory thresholds. In practice, however, it is difficult to adopt this methodology given the time needed to carry out the test, and because audiometry is a subjective exam that requires attention and collaboration from the patient.

In Groups 1 and 2 auditory thresholds increased in proportion to the frequency. This finding was also reported by Zislis,^21^ Green et al.,^42^ Frank,^28^ Hallmo, Borchgrevink & Mair,^43^ Fouquet,^30^ Mattews et al.,^44^ Sakamoto et al.,^31^ Azevedo & Iorio,^32^ Pedalini & Sanchez,^23^ Carvallo et al.,^33^ and Sahyeb, Costa Filho & Alvarenga.^34^

Margolis, Saly & Hunter^10^ also reported sloping audiometric profiles, similar to our results. Other papers corroborate these findings in cases presenting a history of otitis media with effusion.^6^,^7^,^36^,^39^,^37^,^48^

**Chart 1:**
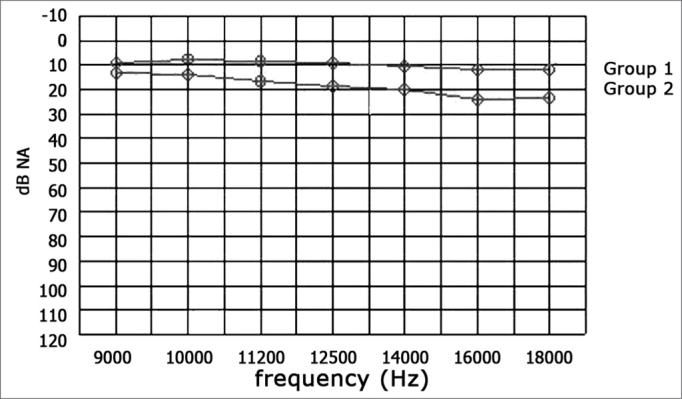
Mean values of auditory thresholds (dB NA) at 9000 to 18000Hz, right ears, groups 1 and 2.

**Chart 2:**
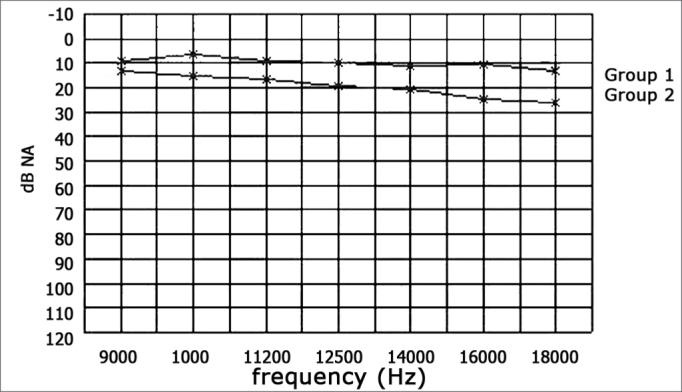
Mean values of auditory thresholds (dB NA) at 9000 to 18000Hz, left ears, groups 1 and 2.

There was a statistically significant difference in auditory thresholds between right and left ears in Group 2 children compared to those in Group 1 at all the frequencies we tested (9000, 10000, 11200, 12500, 14000, 16000, and 18000Hz) (Tables 7 and 8) (Charts 1 and 2). In the literature^5^,^6^,^49^,^39^ various authors have found statistically significant differences in high frequency auditory thresholds between experimental groups and controls; in these studies the experimental groups consisted of children with at least four documented episodes of otitis media with effusion during.

**Tabela 7:** Limiares de audibilidade (dB NA) para as freqüências de 9.000 a 18.000Hz, orelhas direitas entre os dois grupos estudados (valores mostrados como média ± desvio padrão [mediana]).

Freqüências	Grupo 1	Grupo 2
9.000	9,3 ± 2,7 [10]	13,2 ± 4,7 [15]
10.000	7,5 ± 3,3 [10]	13,8 ± 4,5 [15]
11.200	8,2 ± 3,7 [10]	16,5 ± 4,2 [15]
12.500	8,9 ± 4,0 [10]	18,5 ± 4,9 [20]
14.000	10,4 ± 3,7 [10]	20,3 ± 5,1 [20]
16.000	12,1 ± 3,2 [12,5]	24,1 ± 7,5 [25]
18.000	11,8 ± 2,5 [10]	23,5 ± 5,5 [25]

**Table 8:** Auditory thresholds (dB NA) at 9000 to 18000Hz for left ears in both groups (values shown as the mean □ standard deviation [median]).

Frequency	Group 1	Group 2
9.000	8,9 ± 2,9 [10]	12,9 ± 4,7 [15]
10.000	6,4 ± 3,6 [5]	15,3 ± 3,3 [15]
11.200	8,9 ± 3,5 [10]	16,8 ± 3,9 [15]
12.500	9,6 ± 3,7 [10]	19,1 ± 4,0 [20]
14.000	11,4 ± 3,6 [10]	20,6 ± 5,6 [20]
16.000	10,7 ± 3,3 [10]	25,0 ± 7,3 [25]
18.000	13,2 ± 3,2 [15]	26,2 ± 6,5 [25]

These findings may be explained by bacterial toxin and cytotoxin diffusion from the middle ear into the cochlea through the round window.^11^,^12^ Resulting ultrastructural injury in the inner ear, such as rupture of cochlear membranes,^13^,^14^ increases high frequency auditory thresholds.

A further significant point is that otitis media habitually is found in less economically favored societies. Thus, learning disorders that tend to be found in this social class may also result from language disorders and auditory disabilities as a consequence of sensory deprivation and cochlear damage.

Given the results presented in this paper, pediatricians, otorhinolaryngologists, speech therapists, and teachers should be alert for children that present multiple episodes of otitis media. It is important to ask parents questions about the progress of language acquisition and development in the child and to inquire of teachers about the school performance of a given pupil. Many of these children will possibly require pedagogic monitoring and speech therapy together with the medical treatment (antibiotics and middle ear surgery).

## CONCLUSION

Our statistical analysis of the data in this study, which aimed to compare high frequency auditory thresholds in children with the number of episodes of otitis media with effusion, led us to the following conclusions:

1 - There was no statistically significant difference between right and left ear auditory thresholds in both groups at all the frequencies we tested (9000, 10000, 11200, 12500, 14000, 16000, and 18000Hz).
2 - There was a statistically significant difference between right and left ear auditory thresholds in Group 2 subjects compared to Group 1 at all the frequencies we tested (9000, 10000, 11200, 12500, 14000, 16000, and 18000Hz).

- Group 2 had higher angular coefficients compared to Group 1, suggesting elevation of auditory thresholds at higher frequencies.
- High frequency audiometry was able to group separately subjects presenting otitis media with effusion, showing that four episodes of otitis media are statistically enough to significantly alter high frequency auditory thresholds.
